# Supplementary material for: Metagenomic binning with assembly graph embeddings
Source: Bioinformatics. 2022 Aug 16;38(19):4481–7. doi: 10.1093/bioinformatics/btac557 (PMC9525014; doi:10.1093/bioinformatics/btac557)
Supplement: btac557_supplementary_data [file btac557_supplementary_data.zip › amber outputs.zip!/amber outputs/simHC/genome/avg_purity_completeness_bp.pdf]

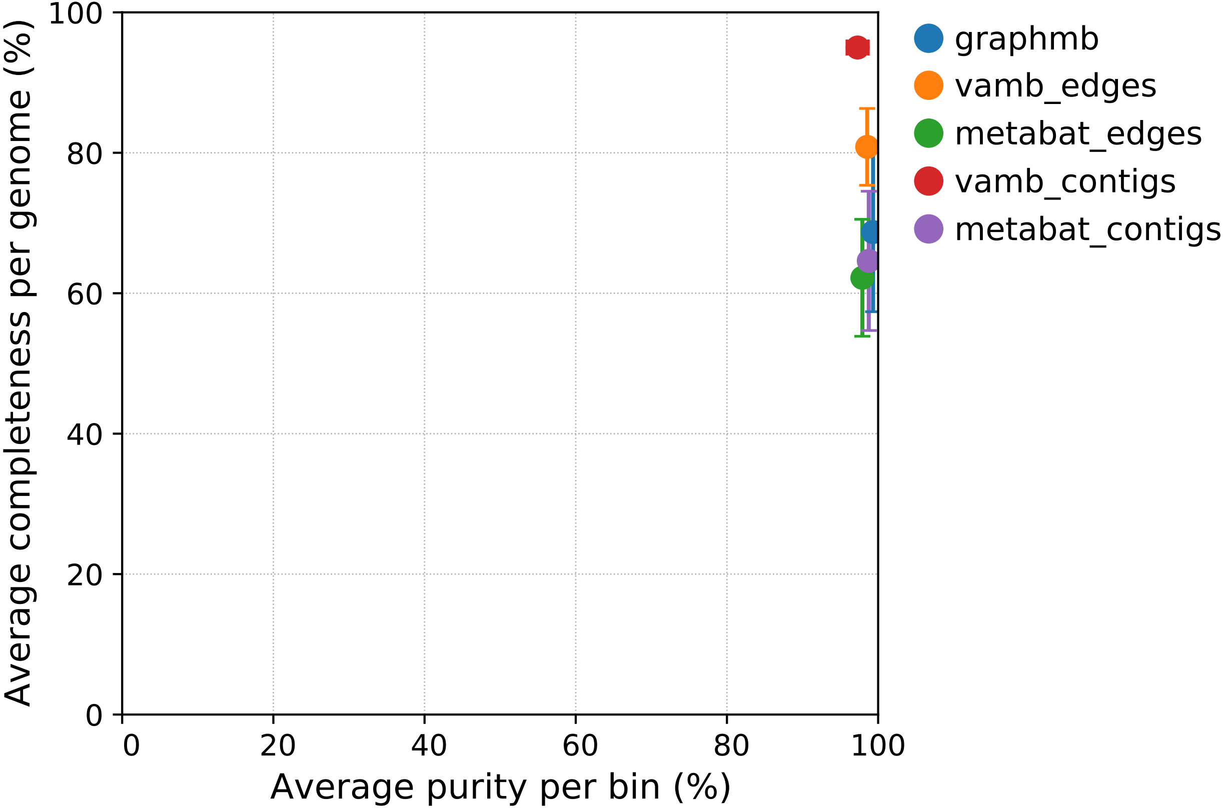

Average completeness per genome (%)
Average purity per bin (%)
0
20
40
60
80
100
0
20
40
60
80
100
graphmb
vamb_edges
metabat_edges
vamb_contigs
metabat_contigs
